# Does Brief Telephone Support Improve Engagement With a Web-Based Weight Management Intervention? Randomized Controlled Trial

**DOI:** 10.2196/jmir.3199

**Published:** 2014-03-28

**Authors:** Laura Dennison, Leanne Morrison, Scott Lloyd, Dawn Phillips, Beth Stuart, Sarah Williams, Katherine Bradbury, Paul Roderick, Elizabeth Murray, Susan Michie, Paul Little, Lucy Yardley

**Affiliations:** ^1^University of SouthamptonSouthamptonUnited Kingdom; ^2^Redcar and Cleveland Borough CouncilRedcarUnited Kingdom; ^3^Durham County CouncilDurhamUnited Kingdom; ^4^Bournemouth UniversityBournemouthUnited Kingdom; ^5^University College LondonLondonUnited Kingdom

**Keywords:** weight loss, obesity, Internet, adherence, behavioral, randomized controlled trial

## Abstract

**Background:**

Recent reviews suggest Web-based interventions are promising approaches for weight management but they identify difficulties with suboptimal usage. The literature suggests that offering some degree of human support to website users may boost usage and outcomes.

**Objective:**

We disseminated the POWeR (“Positive Online Weight Reduction”) Web-based weight management intervention in a community setting. POWeR consisted of weekly online sessions that emphasized self-monitoring, goal-setting, and cognitive/behavioral strategies. Our primary outcome was intervention usage and we investigated whether this was enhanced by the addition of brief telephone coaching. We also explored group differences in short-term self-reported weight loss.

**Methods:**

Participants were recruited using a range of methods including targeted mailouts, advertisements in the local press, notices on organizational websites, and social media. A total of 786 adults were randomized at an individual level through an online procedure to (1) POWeR only (n=264), (2) POWeR plus coaching (n=247), or (3) a waiting list control group (n=275). Those in the POWeR plus coaching arm were contacted at approximately 7 and 28 days after randomization for short coaching telephone calls aimed at promoting continued usage of the website. Website usage was tracked automatically. Weight was assessed by online self-report.

**Results:**

Of the 511 participants allocated to the two intervention groups, the median number of POWeR sessions completed was just one (IQR 0-2 for POWeR only, IQR 0-3 for POWeR plus coach). Nonetheless, a substantial minority completed at least the core three sessions of POWeR: 47 participants (17.8%, 47/264) in the POWeR-only arm and 64 participants (25.9%, 64/247) in the POWeR plus coaching arm. Participants in the POWeR plus coaching group persisted with the intervention for longer and were 1.61 times more likely to complete the core three sessions than the POWeR-only group (χ^2^_1_=4.93; OR 1.61, 95% CI 1.06-2.47; n=511). An intention-to-treat analysis showed between-group differences in weight loss (*F*_2,782_=12.421, *P*<.001). Both intervention groups reported more weight loss than the waiting list control group. Weight loss was slightly, but not significantly, greater in the POWeR plus coaching group. A large proportion of participants assigned to POWeR plus coaching refused phone calls or were not contactable (57.9%, 143/247). Exploratory analyses identified health and sociodemographic differences between those who did and did not engage in coaching when it was made available to them. Users who engaged with coaching used the intervention more and lost more weight than those who did not.

**Conclusions:**

In common with most Web-based intervention studies, usage of POWeR was suboptimal overall. However, our findings suggest that supplementing Web-based weight management with brief human support could improve usage and outcomes in those who take it up.

**Trial Registration:**

International Standard Randomized Controlled Trial Number (ISRCTN): 98176068; http://www.controlled-trials.com/ISRCTN98176068 (Archived by WebCite at http://www.webcitation.org/6OKRjM2oy).

## Introduction

### Background

Internationally, obesity is one of the biggest public health concerns [1]. Interventions that promote changes in diet and physical activity and include behavior modification techniques such as goal setting and self-monitoring are considered the gold standard of treatment [2]. However, high cost and low access limit the reach of such programs when delivered face-to-face by health professionals [3,4]. The Internet has emerged as a promising way to reach greater numbers of individuals at low cost and, in recent years, various Web-based weight loss programs have been developed and evaluated [4-10].

Despite holding promise as potentially cost-effective interventions, recent reviews of Web-based weight loss interventions have found that effect sizes for weight loss tend to be fairly modest, with substantial heterogeneity in outcomes, and many online programs suffer from suboptimal engagement [11-13]. Such findings are not limited to Web-based weight loss interventions but are common across different types of eHealth interventions.

One possible explanation for variations in the efficacy of and engagement with Web-based interventions is the variation in the human contact participants have to support them as they participate in the Web-based program. Human support may be in various formats including face-to-face individual or group meetings, telephone calls, text messages, emails, or online chat. It may be from health professionals, researchers, or technicians and may serve various purposes ranging from answering technical queries, to encouraging prolonged use, to providing substantial therapeutic input. Taken as a whole, the eHealth literature suggests that engagement and behavioral or health outcomes for Web-based interventions tend to be better when usage is accompanied by some form of human contact [14,15]. For example, within the Web-based mental health literature, meta-analyses show larger effect sizes for interventions that also include some contact with a therapist than interventions that are wholly Web-based [16,17], and qualitative studies suggest some participants perceive a need for human contact and support [18,19]. In the field of physical health and weight management, reviews have identified contact, counselling, and support from a health professional as key elements responsible for high engagement and effectiveness of Web-based interventions [20,21]. Interestingly though, the few randomized controlled trials (RCTs) of weight management programs that have directly compared different types or intensities of human support have not always found evidence that higher support versions have superior engagement or weight-related outcomes [4-6]. Yet, in these trials, even participants in the ostensible minimally-supported website arms actually received considerable human contact and support, including initial orientation to the website and contact with the research team throughout the trial [4,5] and counsellor-facilitated online chatrooms [6]. Therefore, these trials cannot provide clear comparisons between Web-based interventions provided with and without human support. Furthermore, recent reviews have drawn attention to how the mixture of modalities and features in Web-based interventions complicates the task of teasing out the impact of any specific component within and across research studies [22].

Overall, there has been insufficient research focused on how to use human contact to boost engagement with Web interventions. This is an important research topic since extensive reach and low marginal cost per additional user are among the key proposed benefits of Web-based interventions [13]. Despite this, many Web-based weight management interventions evaluated to date have featured face-to-face orientation sessions plus various forms of telephone, email, Web chat, or face-to-face contact with a health care professional or researcher during the intervention period [4-8]. By adding human support, the cost and reach benefits may be undermined because costs increase when staff are required, particularly if support is provided by highly trained professionals. Gaining a better understanding of what types of human contact boost engagement with Web interventions, and investigating brief and low-cost formats for such support, is therefore of great importance. It is equally important to understand how human contact/support might influence engagement with and effectiveness of Web interventions. A clear framework for this has been absent until recent theoretical work on “Supportive Accountability”’ [14], which proposes that human support can enhance adherence to eHealth interventions through accountability to another person. Accountability is the expectation that an individual may be called upon to explain his or her actions to another person. The model hypothesizes that successfully fostering “Supportive Accountability” involves some human presence, either face-to-face, or remotely. The model considers progress monitoring to be central to fostering accountability and proposes ways in which to conduct this in an effective and acceptable way. A recent trial using telephone coaching based upon this model showed that this form of human support increased adherence to a Web-based depression intervention [23].

### Current Study Context and Aims

In the current study, we disseminated “POWeR” (Positive Online Weight Reduction), a completely automated Web-based weight management intervention (described in detail below). Other RCTs (ISRCTN31685626 and ISRCTN21244703) are examining the efficacy of POWeR for weight loss in a primary care setting with nurse support. In contrast, the current study sought to investigate engagement with this intervention in a high-reach, low-cost public health context. Unlike previous Web-based weight management trials, our research procedures were handled automatically by our intervention software, which meant that the trial took place without participants having contact with the researchers at registration, baseline, and follow-up. We examined engagement with the intervention and self-reported weight change in this more remote context and tested whether the provision of brief human support influenced this.

Our primary aim was to assess whether human support in the form of brief telephone coaching, based around the Supportive Accountability framework [14], improved engagement with our Web-based weight intervention, as measured by session usage. Secondary analyses examined the self-reported weight loss that participants experienced when following the coached and uncoached version of the intervention. We also explored the uptake of telephone coaching, whether this was associated with user characteristics and outcomes, and whether accountability to a coach might be a mechanism through which coaching boosts engagement with the website and weight loss.

## Methods

### Recruitment

Ethics and research governance approvals were granted by the University of Southampton and the trial was registered (ISRCTN98176068). A variety of methods were used to recruit participants from community settings in the North East of the United Kingdom between June 2012 and January 2013. We mailed out written invitations to 15,000 homes, which resulted in 287 registrations—a 1.91% response rate. Other recruitment methods included local press releases, posters in community settings, and information on local government and NHS (National Health Service) public health websites and intranet, as well as paid advertising on Facebook and posts/tweets on organizational social media.

Recruitment materials invited members of the public to try a new online weight management program as part of a research trial. Recruitment materials and participant information sheets carried the organization name and logo of the local NHS public health organization and also emphasized the involvement of academics and clinicians from the University of Southampton in the development of the intervention. Participation was free and no financial incentives were provided.

### Eligibility, Screening, Consent, and Registration

All recruitment procedures including study information, eligibility screening, obtaining informed consent, baseline data collection, and randomization were conducted online using automated procedures. To proceed through the registration process, participants had to report being UK-resident adults with a body mass index (BMI) of ≥23 and having regular Internet access. Users were cautioned to consult a health professional prior to using POWeR if they reported having a condition that might make changing diet and exercise inappropriate.

The first author’s email address was provided for asking questions prior to signing up (no questions were received). A “POWeR” email address was provided once participants were in the trial. Brief email contact between participants and the first author took place if participants needed to report technical problems or request withdrawal or cancellation of automatic email prompts or reminders. With the exception of coach phone calls and emails received by the POWeR plus coaching participants, there was no other human contact with participants while they were in the trial.

### Randomization and Blinding

Randomization was at the individual level and stratified by BMI (lower BMI <27.5 vs higher BMI ≥27.5) to ensure that the arms were reasonably balanced in terms of overweight, obese, and morbidly obese participants. Participants were allocated with a balanced ratio to one of three arms. The “POWeR only” arm was granted immediate access to the POWeR intervention. The “POWeR plus coaching” arm was granted immediate access to the intervention plus telephone coaching (described below). The waiting list “Control” arm was blocked from using POWeR for 8 weeks. They were not given specific instructions to abstain from weight management or avoid using other interventions during this time. At the end of the 8 weeks, they were provided with access to POWeR (without coaching). It was impossible to blind participants or coaches to trial arm assignment. Researchers were not blinded but did not interact with or collect data from participants directly, as usage was tracked automatically and self-report data was collected via online questionnaires.

### Sample Size

The sample size was calculated a priori using GPOWER v3.1 [24]. We powered the study to compare the POWeR only and the POWeR plus coaching arm on our primary outcome variable (intervention usage). We calculated that we would need 253 participants in each arm to detect a small effect size (*d*=.25) with .80 power (alpha=.05, 2-tailed test). Therefore, we aimed to randomize a total of 759 participants (2 intervention arms and 1 control arm). Because our primary outcome was automatically logged by our website software for all participants, we did not deliberately over-recruit in order to allow for losing participants to follow-up.

### The Web-Based Intervention

POWeR is a fully automated, tailored, Web-based weight management intervention constructed using the LifeGuide open access intervention authoring software [25]. The intervention aims to empower users to become their own personal health trainer through the development of new self-regulation skills. POWeR draws on various theoretical models and incorporates multiple behavior change techniques. The intervention planning and considerable iterative qualitative work undertaken during development stages are described in detail elsewhere [26]. POWeR is structured as a series of online sessions. In the first session, users choose an eating plan, explore their personal motivations for weight loss, and set personalized eating goals to follow in the subsequent week. Further sessions all begin with a weight and goal review (ie, self-monitoring and goal-setting with tailored feedback) and then progress to new content that includes information and tools to help develop cognitive and behavioral self-regulatory weight management skills, each with an explicit scientific rationale. There are interactive activities for participants to complete, user stories/testimonials, and optional links to more detailed information, including reputable external websites. The second session theme is social support and the third focuses on physical activity. The first three sessions are “core” sessions that each user is funneled through; then from the fourth session onward, after initially working through the weight and goal review, users have a choice of whether to also access Web pages about specific topics that interest them (eg, emotional eating, fitting healthy behavior into busy lives) or whether to end the session at that point. A demonstration version of POWeR can be accessed at [27]. See Multimedia Appendix 1 for screenshots.

Intended use of POWeR is the completion of one session per week. Each time a session is completed the subsequent session becomes available 7 days later and remains available until the user next logs in. Participants received automatic email reminders to advise them that their new session is ready, provide a description of what will be covered, and invite them to log in to use it. They also received one automatic email reminder one week later if they had not logged on. A total of 12 different sessions are available and users can continue to complete sessions for as long as they are finding it useful and log in to complete weekly weight and goal reviews even after all sessions have been completed. In the current trial, we followed up with participants and examined their engagement with the intervention and weight loss 8 weeks after randomization.

During the trial, the intervention content was “frozen” and no changes or bug fixes were made to the POWeR website.

### Coaching

The coaching calls aimed to promote continued usage of the POWeR website and adherence to the recommendations within the website. Coaches were postgraduate students and research assistants affiliated with the health psychology research center at the University of Southampton who had been provided with training in the coaching procedures and a brief introduction to the POWeR website. Coaching procedures were developed based on the Supportive Accountability model [14]. Coaches could access a coaching portal of POWeR where they were able to review the usage patterns of participants, a graph showing weight change, and the participant’s current eating and physical activity goals and plans. Coaching sessions were focused on promoting on-going use of the Web intervention by monitoring usage and giving feedback on progress, and offering support and encouragement for use of the website. The calls were scheduled for one week and four weeks after participants were granted access to POWeR. Each coaching call was intended to last for approximately 10 minutes. Coaches followed detailed protocols that set out ways to proceed with the two phone calls depending on whether the user was engaging with POWeR as intended (summarized in Table 1). Multimedia Appendix 2 contains detailed coaching protocols, plus further details on scheduling of coaching sessions, attempts made to contact participants, and the coaches’ background, training, and supervision.

**Table 1:** Summary of content of coaching telephone calls.

Call	Content
**Coaching call 1 (week 1)**	
	Welcome participant to POWeR
	Build a friendly relationship
	Explain what the role of the coach is/is not
	Explain how progress monitoring will be conducted and reassure that it will be done in a supportive and encouraging way
	Review POWeR use so far (with reference to data available in the coach portal)
	Praise/encourage any POWeR use (or gently explore reasons for non-use and encourage future use)
	Ask about questions and concerns and point in direction of POWeR tools/future sessions
	Ask about eating goals and plans (with reference to data available in the coach portal) and offer encouragement
	Remind about on-going monitoring and another phone call in week 4
**Coaching call 2 (week 4)**	
	Build a friendly relationship
	Remind about reason for today’s call
	Review POWeR use (with reference to data available in the coach portal)
	Praise/encourage any POWeR use (or gently explore reasons for non-use and encourage future use).
	If relevant, congratulate on weight loss (with reference to data available in the coach portal)
	Ask about questions and concerns and point in direction of POWeR tools/future sessions
	Ask about eating and physical activity goals and plans (with reference to data available in the coach portal) and offer encouragement
	Mention coaching is ending and suggest considering support from elsewhere

### Measures and Data Collection

The primary outcome variable (usage of the Web-based intervention) was automatically logged by the intervention software. The LifeGuide software logs all usage data including which pages were viewed, in what order, when, and for how long. For the current analyses, we analyzed the number of POWeR sessions each participant had completed by 8-week follow-up.

All self-report data were collected using Web-based questionnaires. To ensure we had complete data on our participants at baseline, all baseline questionnaires were mandatory (ie, the participant could not progress without submitting a response). The follow-up point was 8 weeks post-randomization. Automatically generated emails requested participants to complete a brief follow-up questionnaire and included a hyperlink to Web-based questionnaires. Up to three reminder emails were automatically issued after 5, 10, and 15 days of non-response.

At baseline, we collected demographic data including: age, gender, marital status, ethnicity, highest education level, employment status, postcode (from which we derived an Index of Multiple Deprivation [IMD]), health literacy (a single-item measure) [28], and estimated weekly hours of Internet usage. Participants self-reported height (in cm or feet/inches) and weight (as measured on home scales). Participants also reported whether a health professional had ever advised weight loss or referred them to weight management programs, and whether they had asthma, diabetes, heart disease, hypertension, or a stroke.

At follow-up, participants in all treatment arms were asked to enter their current weight and whether they had followed any other weight loss programs over the last 8 weeks. Participants in both of the active treatment arms were also administered the Supportive Accountability Questionnaire [29], which assesses the user’s perceptions that they are held accountable to somebody else for their adherence to the intervention. Participants indicated on a 7-point Likert scale the extent to which they agreed with six statements. For coach arm participants, the items referred to a coach (eg, “I believe my POWeR coach is aware of and notices when I use the website”) and for Web only participants, questions referred instead to “the POWeR team”. Higher scores indicated higher perceived accountability (Cronbach alpha=.70). Uptake of the two available coaching sessions was recorded by the coaches.

### Analysis

#### Overview

All analyses were conducted in SPSS version 20. Means and standard deviations were computed for continuous variables, and “n” and percentage were computed for categorical variables. We used an alpha level of .05 for all statistical tests.

#### Primary Outcome: Intervention Usage and Between-Arm Differences

For the primary analysis, we planned to conduct independent *t* tests to examine between-arm differences in mean number of sessions completed. However, due to highly skewed data, such analysis was inappropriate. Instead, and as recommended by Glasgow et al [5], we computed a meaningful “threshold” usage dichotomous variable that indicated whether or not the participant had completed the core three POWeR sessions. Between-arm differences were then analyzed using a chi-square test.

#### Secondary Outcome: Self-Reported Weight Loss and Between-Arm Differences

To examine our between-arm differences in self-reported weight loss, we used ANCOVA (analysis of covariance), with follow-up weight as a dependent variable, baseline weight as a covariate, and trial arm as the independent variable. We performed an intention-to-treat (ITT) analysis. Where weight at follow-up was missing, this was imputed using the “Multiple Imputation” procedure in SPSS. We performed 100 imputations using baseline variables and any available weight measurements as predictors. ANCOVA was performed and the pooled results from the multiple imputation reported. We also conducted a completers analysis by repeating another ANCOVA on the sample of participants who had completed follow-up measures. We also categorized participants according to whether or not they had lost at least 3 kg at 8-week follow-up. Such weight loss would correspond to approximately 0.4 kg (just under 1 lb) weight loss per week and would indicate a rate of weight loss in line with the POWeR program recommendations, which emphasize building healthy habits rather than rapid weight loss. We reported the percentage of participants in each arm meeting this criterion.

We produced descriptive statistics to summarize coaching uptake and used *t* tests or chi-square tests to explore whether coach participants who had the full dose of coaching (ie, both sessions) differed from those who did not on baseline variables, sessions completed, weight loss, and Supportive Accountability.

## Results

### Participants

**Figure 1:**
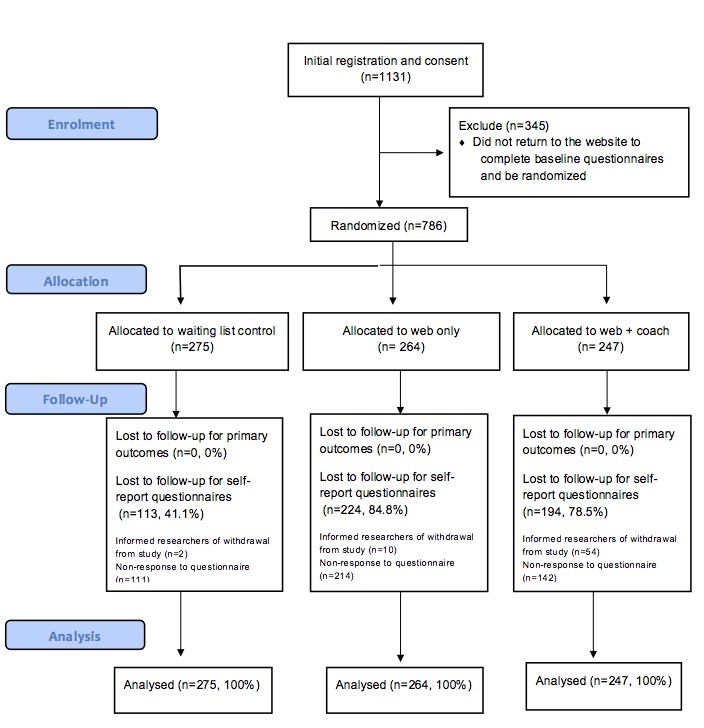
Flow of participants through the trial.

Figure 1 shows flow of participants through the study. Between June 2012 and January 2013, 1131 users completed the initial step in the registration process. Of these, 786 (69.50%) subsequently returned to the website, completed the baseline questionnaires, and were randomized. A total of 275 participants (35.0%) were randomized to the control arm, 264 to POWeR only (33.6%), and 247 (31.4%) to POWeR plus coaching.

The primary outcome, website usage, was successfully tracked for 100% of randomized participants. However, loss to follow-up was very high for the self-report measures. Full or partial self-report follow-up data at 8 weeks was provided by only 58.9% (162/275) of control, 15.2% (40/264) of POWeR only, and 21.5% (53/247) of POWeR plus coach participants. A total of 246 participants provided weight data at follow-up; 540 did not. Chi-square tests showed that missingness of weight data at 8 weeks was related to trial arm, with control participants more likely to provide data than participants in the two intervention arms. Looking within the two intervention arms, missingness was also related to website usage, with those having used POWeR the most (≥3 sessions) being more likely to provide follow-up data than those with lower usage (<3 sessions). Most baseline demographic, health, or weight-related variables were unrelated to missingness but participants who were older, less deprived, and university educated were more likely to provide follow-up data. All baseline variables, including those that were significantly associated with failure to provide follow-up data, were included as predictors in the multiple imputation model.

### Participant Characteristics

Table 2 shows demographic and health characteristics of the sample. Participants were predominantly female, white British, and around half (444/786, 56.5%) were married. The age range of users was wide, with a mean of 44 years (SD 12.7). Overall, participants tended to live in fairly non-deprived areas, although scores ranged from deprived to fairly affluent. Few participants had problems with health literacy. Participants were mostly overweight and obese, with 15.0% (118/786) meeting criteria as morbidly obese and a minority (48/786, 6.1%) falling into the top end of the normal/healthy weight category. One-third (276/786, 35.1%) reported obesity-related health conditions (hypertension, diabetes, asthma, heart disease, or stroke). Roughly half (417/786, 53.1%) reported ever having been advised to lose weight by a health professional, around one-fifth (163/786, 20.7%) had ever been referred to a weight management program by a health professional, and just under half (383/786, 48.7%) said that they were currently or recently engaged in weight management attempts. One-way ANOVA (analysis of variance) and chi-square tests revealed no significant differences between groups at baseline on any of the sociodemographic, health, or weight-related variables.

**Table 2:** Participant characteristics.

Characteristic		Full sample (n=786)	Control (n=275)	POWeR only (n=264)	POWeR plus coaching (n=247)
Age in years, mean (SD)		44.0 (12.7)	44.2 (13.0)	43.3 (12.5)	44.4 (12.6)
Female gender, n (%)		628 (79.9%)	216 (78.5%)	217 (82.5%)	195 (78.9%)
White British ethnicity, n (%)		760 (96.7%)	265 (96.4%)	253 (95.8%)	242 (98.0%)
**Marital status, n (%)**					
	Married	444 (56.5%)	154 (56.0%)	147 (55.7%)	143 (57.9%)
	Living with partner	120 (15.3%)	41 (14.9%)	37 (14.0%)	42 (17.0%)
	Single	123 (15.6%)	44 (16.0%)	44 (16.7%)	35 (14.2%)
	Divorced or separated	78 (9.9%)	32 (11.7%)	22 (8.4%)	24 (9.7%)
	Widowed	15 (1.9%)	2 (0.7%)	10 (3.8%)	3 (1.2%)
**Highest education, n (%)**					
	No formal	42 (5.3%)	16 (5.8%)	11 (4.2%)	15 (6.1%)
	GCSE^a^or equivalent	178 (22.6%)	60 (21.8%)	62 (23.5%)	56 (22.7%)
	A levels or equivalent	110 (14.0%)	35 (12.7%)	39 (14.8%)	36 (14.6%)
	University (undergraduate or postgraduate)	253 (32.2%)	98 (35.7%)	86 (32.6%)	69 (27.9%)
	Diploma / professional / vocational qualification	197 (25.1%)	64 (23.6%)	63 (23.8%)	69 (27.9%)
**Employment status, n (%)**					
	Full or part time employment / self-employment	555 (70.6%)	189 (68.6%)	184 (69.8%)	182 (73.6%)
	Not working due to sickness or disability	22 (2.8%)	6 (2.2%)	7 (2.7%)	9 (3.6%)
	Unemployed	20 (2.5%)	7 (2.5%)	7 (2.7%)	6 (2.4%)
	Homemaker	31 (3.9%)	14 (5.1%)	11 (4.2%)	6 (2.4%)
	Student	65 (8.3%)	21 (7.6%)	26 (8.9%)	18 (7.3%)
	Retired	75 (9.5%)	33 (12.0%)	21 (8.0%)	21 (8.5%)
IMD^b^score (higher is more deprived), mean (SD)		25.9 (15.3)	25.2 (14.5)	26.4 (15.5)	26.0 (16.0)
Health literacy (1-5, higher is poorer literacy), mean (SD)		1.1 (0.4)	1.1 (0.4)	1.1 (0.4)	1.1 (0.4)
Internet usage (typical hours per week), mean (SD)		13.0 (12.0)	13.6 (13.6)	13.0 (10.7)	12.4 (11.5)
BMI^c^, mean (SD)		33.0 (7.0)	32.9 (6.8)	33.1 (6.4)	33.1 (7.8)
**BMI category, n (%)**					
	Upper part of normal / healthy range (23-24.9)	48 (6.1%)	18 (6.5%)	17 (6.4%)	13 (5.3%)
	Overweight (25-29.9)	267 (34.0%)	100 (36.4%)	80 (30.3%)	87 (35.2%)
	Obese (30-39.9)	353 (44.9%)	111 (40.4%)	128 (48.5%)	114 (46.2%)
	Morbidly obese (40+)	118 (15.0%)	46 (16.7%)	39 (14.8%)	33 (13.4%)
Has one or more of the following health conditions (hypertension, diabetes, heart disease, asthma, stroke)		276 (35.1%)	96 (35.3%)	85 (33.2%)	95 (39.4%)
Ever advised to lose weight by a health professional		417 (53.1%)	150 (55.1%)	141 (54.2%)	126 (52.1%)
Ever referred to a weight management service / program by a health professional		163 (20.7%)	61 (22.3%)	53 (20.5%)	49 (20.6%)
Current / recent attempt to manage weight		383 (48.7%)	128 (46.5%)	136 (51.5%)	119 (48.2%)

^a^GCSE: General Certificate of Secondary Education

^b^IMD: Index of Mass Deprivation score

^c^BMI: Body Mass Index

### Primary Outcome: Intervention Usage and Between-Arm Differences

Website usage patterns were analyzed in the 511 participants allocated to the two active intervention arms. Overall, the number of POWeR sessions completed was low. The median number of sessions completed was 1 in both the POWeR only and the POWeR plus coaching arm (IQR 0-2 for POWeR only, IQR 0-3 for POWeR plus coaching). The data were positively skewed because around one-third of participants (94/264, 35.6% of POWeR only; 80/247, 32.4% of POWeR plus coaching) never completed a session and many participants completed only one or two sessions. Nonetheless, a substantial minority completed at least the core three sessions of POWeR (ie, the meaningful usage threshold) (Table 3). Those in the POWeR plus coaching arm were 1.61 times (95% CI 1.06-2.47) more likely to have continued to use POWeR until at least the end of the core three sessions (χ^2^_1_=4.93; *P*=.026; n=511).

**Table 3:** Usage of POWeR sessions.

Usage	POWeR only (n=264), n (%)	POWeR plus coaching (n=247), n (%)
Did not reach the meaningful usage threshold (<3 sessions)	217 (82.2%)	183 (74.1%)
Reached the meaningful usage threshold (≥3 sessions)	47 (17.8%)	64 (25.9%)

### Secondary Outcome: Self-Reported Weight Loss and Between-Arm Differences

**Figure 2:**
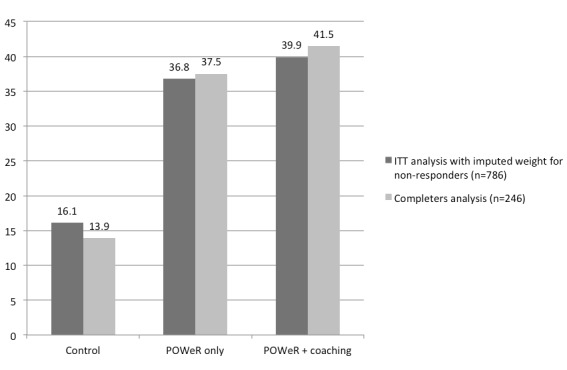
Percentage of participants self-reporting a weight loss of ≥3 kg at 8-week follow-up by treatment arm.

Table 4 shows weight data for the entire sample with follow-up weight imputed for those participants who did not provide data at 8 weeks. Overall, weight loss was highest in the coaching arm and lowest in the control arm. Between-arm differences were significant (*F*_2,782_=34.02, *P*<.001). Post-hoc pairwise comparisons indicated that those in the coaching arm lost more weight than those in the control arm (mean difference 1.97 kg, *P*<.001, *d*=−.63, 95% CI −1.40 to 2.49). Those in the POWeR only arm also lost more weight than those in the control arm (mean difference 1.70 kg, *P*<.001, *d*=−.54, 95% CI 1.15-2.22). The difference between the POWeR plus coaching and the POWeR only arms was not significant (mean difference=0.27 kg, *P*=.676, *d*=−.08, 95% CI −0.29 to 0.81). The proportion of participants losing 3 kg or more was highest in the POWeR plus coaching arm and lowest in the control arm (Figure 2).

Weight data for the 246 follow-up completers is shown in (Table 5). Between-arm differences in weight loss were significant (*F*_2,242_=20.73, *P*<.001). Post-hoc pairwise comparisons indicated that differences were between the control and each of the active treatment arms (mean difference between POWeR only and control 2.49, *P*<.001, *d*=−.82, 95% CI −1.18 to −0.46 and between POWeR plus coaching and control 2.69, *P*<.001, *d*=−.89, 95% CI −1.22 to −0.5567), but not between the POWeR plus coaching and the POWeR only arms (mean difference 0.20 kg, *P*=.755, *d*=−.07, 95% CI −0.49 to 0.35). The proportion of completers losing 3 kg or more was highest in the POWeR plus coaching arm and lowest in the control arm (Figure 2). In order to ensure that the ANCOVA results were not an artefact of an incorrect assumption about the normality of the data, we also conducted a non-parametric analysis using quantile regression and controlling for baseline weight. The results of this approach echoed those of the parametric analysis with a significantly lower median weight in the intervention groups than the control group (*P*=.019 for the POWeR only group and *P*<.001 for the POWeR + coach group) but no statistically significant difference between intervention groups (*P*=.207)

**Table 4:** Weight change by treatment arm (ITT analysis).

Weight	Control (n=275)	POWeR only (n=264)	POWeR plus coaching (n=247)
Weight at baseline (kg), mean (SD)	91.64 (20.31)	92.02 (20.09)	91.86 (20.96)
Weight at follow-up (kg), mean (SD)	91.34 (20.15)	90.00 (19.89)	89.59 (20.65)
Weight change (kg), mean (SD)	−0.30 (2.82)	−2.01 (3.45)	−2.27 (3.41)

**Table 5:** Weight change by treatment arm (follow-up responders only).

Weight	Control (n=158)	POWeR only (n=39)	POWeR plus coaching (n=49)
Weight at baseline (kg), mean (SD)	91.85 (20.42)	90.53 (16.86)	94.80 (23.64)
Weight at follow-up (kg), mean (SD)	91.38 (20.47)	87.67 (16.10)	91.63 (23.17)
Weight change (kg), mean (SD)	−0.41 (2.43)	−2.86 (4.42)	−3.17 (3.61)

### Exploring Coaching Uptake

Overall, uptake of coaching calls was low. More than half (57.9%, 143/247) of those in the POWeR plus coaching arm actually received no coaching calls. Of the 104 participants that had coaching, most (n=58, 55.8%) had just one call. Only 46 participants (18.6%, 46/247) received both calls (ie, a full dose of coaching as per protocol). When coaching calls occurred, they tended to last for roughly 7.5 minutes each. The low rate of coaching calls can be partially explained by participant withdrawal, as 54 (21.9%, 54/247) POWeR plus coaching arm participants withdrew during the 8-week study period (see Figure 1), but the remainder of those who did not receive coaching calls simply did not answer the phone calls.

Compared to POWeR plus coaching participants who did not receive full coaching, participants that had the full “dose” of coaching (ie, both calls) tended to be older (*t*_245_=−4.03, *P*<.001, *d*=−.66) and have higher baseline BMI (*t*_245_=−2.13, *P*=.038, *d*=.35). They were 2.16 times more likely to have hypertension (χ^2^_1_=4.38; *P*=.036; n=241) and 2.79 times more likely to have been referred to a weight loss scheme by a health professional (χ^2^_1_=8.22; *P*=.004; n=238). Table 6 shows that overall, participants allocated to POWeR plus coaching who did not receive full coaching had usage and weight outcomes similar to (or slightly worse than) participants allocated to the Web-only arm. Compared to participants allocated to POWeR plus coaching who did not receive full coaching, those that did receive full coaching completed significantly more POWeR sessions (*t*_55.186_=−5.78, *P*<.001, *d*=.94) and were 6.73 times more likely to have completed the core sessions (χ^2^_1_=31.65 (n=247), *P*<.001). Their mean weight loss was 2.79 kg compared to 0.77 kg (*t*_50.459_=3.82, *P*<.001, *d*=.623) and they were 5.12 times more likely to have lost 3 kg or more (χ^2^_1_=19.75 (n=247), *P*<.001). Supportive Accountability scores were slightly, but not significantly higher between full coaching receivers/non-receivers (*t*_50_=−1.38, *P*=.17, *d*=.38) and between all participants allocated to the coach arm and those in the Web arm (*t*_90_=−1.14, *P*=.256, *d*=.24).

**Table 6:** Differences in usage, weight outcomes, and supportive accountability in the active treatment arms depending on whether full coaching was received.

	POWeR only n=264	POWeR plus coaching n=247	
		Did not receive full coaching n=201	Received full coaching n=46
Number of weekly POWeR sessions completed, median (IQR)	1 (0-2)	1 (0-2)	4 (2-6)
Number of participants completing the 3 core POWeR sessions, n (%)	47 (17.8%)	37 (18.4%)	27 (58.7%)
Weight change (kg), mean (SD)	−0.80 (2.22)	−0.77 (1.78)	−2.79 (3.48)
Participants achieving recommended amount of weight loss, ie, 3 kg or more,n (%)	27 (10.2%)	19 (9.5%)	16 (34.8%)
Supportive Accountability, mean (SD)^a^	3.63 (1.30)	3.72 (1.08)	4.11 (0.94)

^a^Data based on responders to follow-up from both active treatment arms (n=40 in Web only, n=25 who received full coaching, and n=27 who did not receive full coaching).

## Discussion

### Engagement With the POWeR Intervention and Differences Between Groups

In common with most studies of Web-based interventions [9,30], participants overall made suboptimal use of the intervention. However, usage was indeed improved, as predicted, by supplementing the website with brief phone calls focused on improving engagement. Despite the majority of the eHealth literature suggesting that human support is important for boosting engagement and outcomes [16-21], several previous Web-based weight loss trials have found that providing more intense support did not make a significant difference to usage or outcomes [4-6]. One explanation for why our study did identify website usage differences between website plus coaching and website-only participants might be that our website-only condition was (in contrast to previous studies) a completely stand-alone intervention that was registered for and used without any contact with the research team or health professionals. Therefore, we were truly comparing a supported and an unsupported version. Another explanation is that, by explicitly basing our coaching around a theoretical framework that specifically seeks to delineate how to boost adherence and engagement with Web-based interventions, the human support we offered may have been more successful than that in previous studies.

### Self-Reported Weight Loss and Differences Between Groups

Despite the overall pattern of light usage, in our secondary analyses of weight loss we showed that both of our intervention arms reported losing more weight than the control arm. Furthermore, a substantial minority of participants in the POWeR only and the POWeR plus coaching arm had high engagement with website sessions and reported losing clinically important amounts of weight (≥3 kg) at short-term follow-up. Hence, even though the effect sizes of the interventions were small overall, the impact at public health level could be considerable, given the low costs associated with entirely automated (POWeR only) or minimally-supported (POWeR plus coaching) Web-based interventions.

Encouragingly, our exploratory analyses tentatively indicated that the higher engagement seen in the POWeR plus coaching arm may have been associated with improved effectiveness of the Web-based intervention. Differences in weight loss between the POWeR only and the POWeR plus coaching arm were not significant overall, but substantial effect sizes were observed when comparing participants in the coaching arm who actually received both coaching phone calls to those who did not.

### Uptake of Telephone Coaching

The impact of coaching on both usage and weight loss outcomes is likely to have been reduced by low uptake, as less than one in five participants in the coaching arm actually received both phone calls. The reasons for low uptake of coaching are not entirely clear. Some participants might have welcomed the opportunity for coaching but were unreachable by telephone when the calls were attempted—even though coaches made several attempts to contact participants and tried to accommodate their preferred contact times. However, some participants may have found the prospect of coaching off-putting. Indeed, the higher rate of withdrawal from the study observed in the coach arm compared to the website-only arm (n=54 vs n=10) might be taken as an indication that some participants disliked the prospect of coaching. Although most participants did not provide a reason for their withdrawal (typically they simply emailed “please withdraw me from the POWeR study”), we noted that most withdrawals happened shortly after allocation to the coach arm, or around the time participants were expecting the first coaching call. This raises the possibility that offering coaching may have multiple impacts: for some, it may boost usage but for others it might actually increase the likelihood of attrition. Whether certain groups of Web intervention users would be more comfortable with and responsive to human support if it was provided through different channels is an interesting question, requiring further study. It may be that there are groups of users who would cease using the intervention, whatever support was provided, and some who actually prefer to have no human contact and see the privacy and independence associated with Web-based interventions as a benefit.

A positive finding was that uptake of coaching calls was greatest among users who seemed particularly suitable candidates for weight management interventions (ie, more overweight, more likely to have hypertension, and to have been referred to a weight management program by a health professional). Future studies could build on this work to investigate further the users who are most likely to use and benefit from human support for Web interventions and how best to overcome barriers to uptake.

While the current study focused on a telephone-based form of human support, different approaches to boosting engagement have either capitalized on more recent technology or have emphasized peer support as an alternative to a health professional or coach. For example, Web-based health interventions have made use of email contact [10], SMS [10,31], online chatrooms or forums [6,10], and link-ups to online social networking sites [32]. Although some studies suggest these approaches may be promising in boosting usage, engagement, and outcomes, others suggest low uptake and lack of interest from users. Furthermore, these studies were not designed to isolate the influence of these supposed supportive features. We therefore do not yet have a substantial body of systematic research comparing interventions delivered with and without these supportive features and consequently have limited insight into likely uptake, effectiveness, and cost-effectiveness.

### Supportive Accountability as a Mechanism for Boosting Engagement

One of our study objectives was to explore whether coaching, based around the Supportive Accountability model [14], increased participants’ perceived accountability for using the intervention. Although we did not detect a statistically significant between-group difference, this analysis may be underpowered due to high loss to follow-up. Indeed, effect sizes were consistent with a trend toward greater levels of perceived accountability in those who received coaching. Another explanation for not observing substantial between-group differences in accountability is that there may have been a ceiling effect, since perceptions of accountability were moderately high in both the coached and uncoached participants who responded to this questionnaire. The POWeR website itself and/or the context of participating in research may have instilled a certain level of accountability regardless of whether there was any coaching available. POWeR involves weekly self-monitoring and tailored feedback and has many interactive features and a human tone that may have been successful in promoting perceptions of accountability. Although recent eHealth research and reviews have highlighted contact with a human as important for boosting engagement and effectiveness [14,20,21], some people may find it sufficient to use Web-based programs that effectively mimic some of the important aspects of human support, whether that be accountability, being treated as an individual, or feeling that somebody cares. Further research could seek to identify mechanisms through which human support offers additional benefits and explore whether well-designed and engaging website features can successfully simulate some of these features and processes.

### Strengths and Limitations

A strength of this research is that our coaching protocols were well-documented, specific in their aims, and based on a theoretical model of engagement with digital interventions. Such explicit explanation of the aims and nature of human contact is rare in the reporting of Web-based interventions. Furthermore, the coach contact was brief (around 15 minutes for participants receiving the full dose) and delivered by providers with minimal training. This type of additional human support should be replicable in future studies and might prove feasible to implement and cost-effective for improving engagement and boosting intervention effectiveness even if effect sizes are modest.

The current study benefited from having primary outcome data available from all participants (by automatically tracking website usage), allowing this analysis to include all randomized participants. However, our pragmatic research design, which included minimal contact between researchers and participants and which probably attracted participants who were curious but not committed to following an online weight management program, may have contributed to the very high loss to follow-up for the secondary outcome data collected via self-reported questionnaires. Low follow-up rates are common in Web-based intervention trials, especially when research methodologies are more in line with a pragmatic trial than an efficacy trial. However, the large amount of missing data at follow-up limited statistical power and reduced our ability to draw firm conclusions about change and group differences in the self-reported follow-up data. Therefore, our secondary and exploratory analyses based on these measures need to be interpreted with caution.

Due to the large number and wide geographical dispersion of participants, only self-reported weight data could be obtained. Most Web-based weight loss trials have obtained objective weight data at face-to-face baseline and follow-up assessments [6-10]. Although in one respect self-reported weight data is a limitation of this study, on the other hand the absence of face-to-face assessment in this study allowed us to obtain website usage data in a context where there is no contact with a researcher. We believe that weight loss, website usage, and retention for follow-up in many existing Web weight loss trials may be influenced to some degree by the contact with researchers, the expectation of being weighed by the research team at a later date, and the perceptions of accountability and pressure this creates. In the current pragmatically-oriented study, we may have obtained a more representative view of users, usage patterns, and weight loss in contexts similar to what could be practical and affordable for a public health intervention. Furthermore, a recent study suggests that online self-reported weight tends to be reasonably accurate [33]. Nonetheless, we cannot rule out the possibility that contact with the coach in the coaching arm may have influenced participants’ self-reporting of weight in some way, potentially leading to biased (possibly inflated) self-reports of weight loss at follow-up. However, given that testing the efficacy of POWeR for weight loss was not our main research question in this trial, we believe the self-report data remains a useful initial indicator of weight loss outcomes from using POWeR with and without coaching.

This study chose to use the number of sessions completed as the indicator of participant engagement with the intervention. This has several advantages, including that it allowed us to obtain objective data unobtrusively for every participant and gave a reliable indication of the “dose” of the intervention that participants had been exposed to and which aspects they had seen. It is, however, not the only way to usefully investigate participant engagement and only gives a rudimentary picture of the extent to which participants were absorbing, understanding, and applying material presented on the Web pages. Future research may wish to use alternative ways of operationalizing engagement in order to investigate how deeply participants are engaging with intervention content. The challenge facing researchers, however, is how to measure engagement without relying on self-report follow-up data, which in many Web-based trials is unlikely to be provided by the majority of users.

### Conclusions

In common with most Web-based intervention studies, usage of POWeR was suboptimal overall. Our findings suggest that supplementing Web-based weight management with brief human support might have a modest effect on persistence with the Web-based sessions, might improve weight loss outcomes, and could prove cost-effective. However, uptake of telephone support may be low overall, with particular types of users more likely to engage with it. Further research is needed to understand and optimize strategies to keep users engaged with Web-based weight interventions.

## Multimedia Appendix 1

Screenshots from POWeR.

## Multimedia Appendix 2

Coaching protocol.

## Multimedia Appendix 3

CONSORT-EHEALTH checklist V1.6.2 [34].

## Abbreviations

ANCOVA: analysis of covariance
BMI: body mass index
IMD: Index of Multiple of Deprivation
ITT: intention to treat
NHS: National Health Service
POWeR: Positive Online Weight Reduction intervention
RCT: randomized controlled trial
