# Supplementary material for: Does Brief Telephone Support Improve Engagement With a Web-Based Weight Management Intervention? Randomized Controlled Trial
Source: J Med Internet Res. 2014 Mar 28;16(3):e95. doi: 10.2196/jmir.3199 (PMC4004138; doi:10.2196/jmir.3199)
Supplement: Supplementary file 1 [file jmir_v16i3e95_app1.pdf]

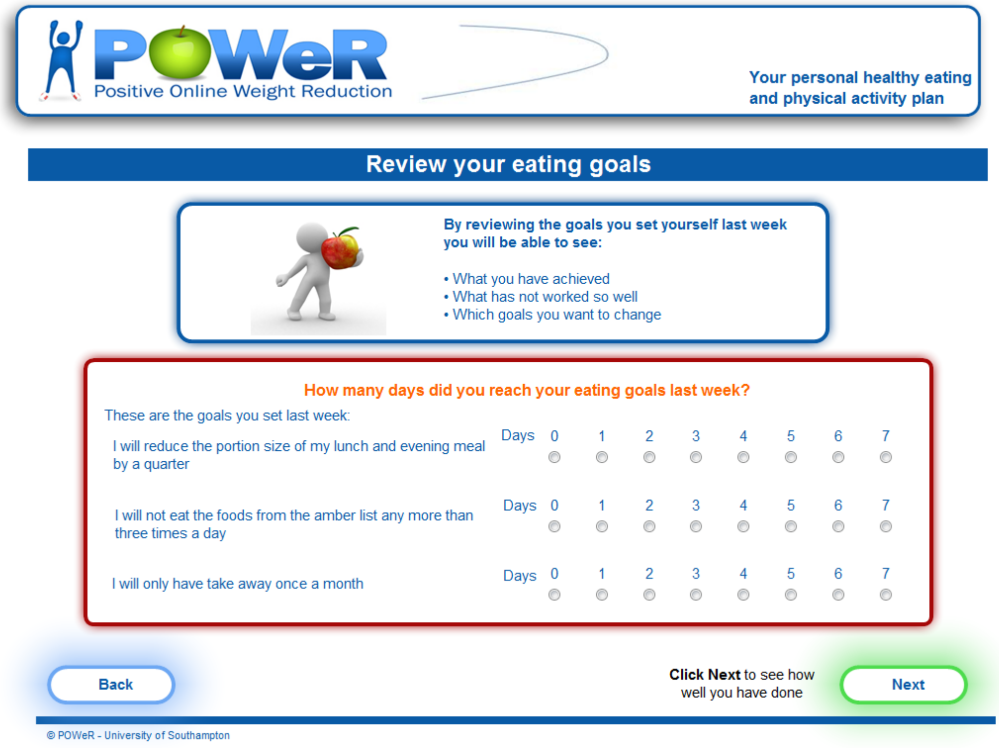

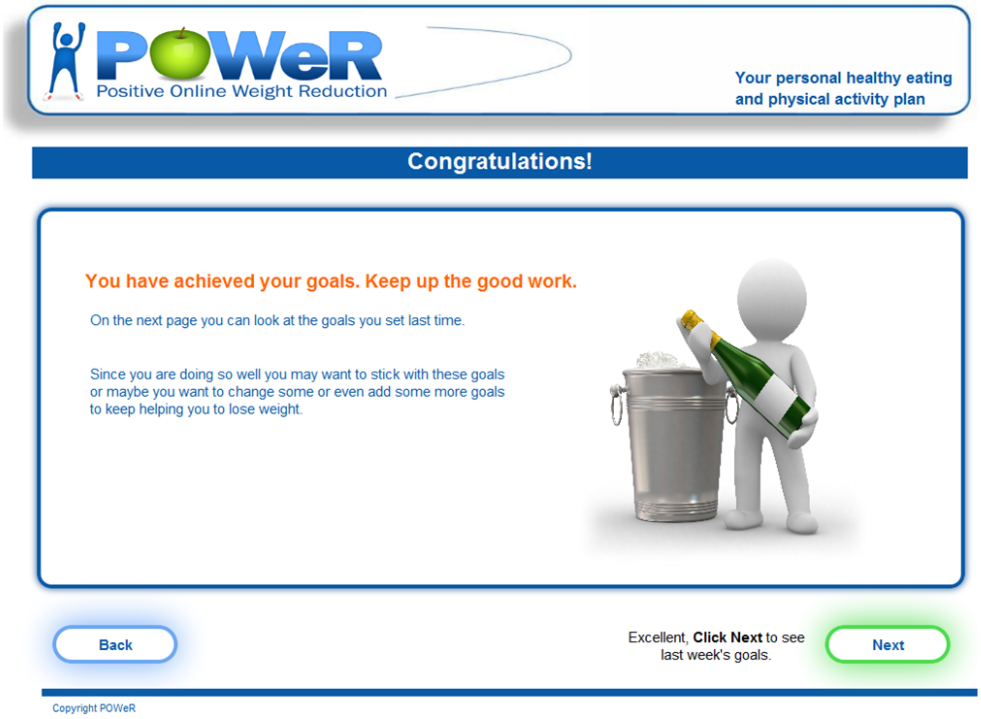

Screenshots from POWeR: goal review and personalised feedback
