# Supplementary material for: Does Brief Telephone Support Improve Engagement With a Web-Based Weight Management Intervention? Randomized Controlled Trial
Source: J Med Internet Res. 2014 Mar 28;16(3):e95. doi: 10.2196/jmir.3199 (PMC4004138; doi:10.2196/jmir.3199)
Supplement: Supplementary file 2 [file jmir_v16i3e95_app2.pdf]

Protocols for conducting telephone coaching for Positive Online Weight Reduction (POWeR) intervention users

Dennison, L., Morrison, L., Lloyd, S., Phillips, D., Stuart, B., Roderick, P., Michie, S., Murray, E., Little, P., Yardley, L. (2014). Does brief telephone support improve engagement with a web-based weight management intervention? A randomised controlled trial. *Journal of Medical Internet Research*

Details of coaches background, training and supervision

The coaches were eight postgraduate students and research assistants affiliated with the health psychology research centre at the University of Southampton. Assignment to a particular coach was on the basis of coach workload/availability. With one exception (where the coach left the institution), all participants had the same coach for both calls.

Coaches completed approximately 3.5 hours of training each. This included working through the core sessions of POWeR themselves and attending a training session which covered trial procedures, the POWeR intervention philosophy and the coaching protocol.

Coaches were closely supervised throughout the trial period by the first author who listened to audio recordings of the coaching sessions and used a brief checklist to help monitor and ensure fidelity to protocol. Any departures from protocol were discussed promptly with the coach.

Coaching procedures and scheduling

Following randomisation, participants in the coach arm were shown a web page advising them that they were going to be contacted by a POWeR coach who would provide them with support in following the programme. The web page allowed participants to suggest optimal times/days for being contacted (limited to weekdays 8am to 8pm) and provide details of the best phone numbers for coaches to contact them on.

Attempts to contact participants for their coaching calls were made at the end of week 1 and the end of week 4. Decisions about optimal scheduling of coaching calls were based on a combination team’s previous experience with piloting POWeR, clinical experience, and the tendency for e-health studies to report large discontinuation rates with substantial drop-out in early sessions. We limited the number of coaching sessions to two in order to attempt to test a relatively low intensity, low cost form of human support. We chose to have the first coaching session at

week 1 (7 days after access to it was granted) as we expected that for those who had already done session 1 of POWeR this would encourage subsequent weekly log-ins and for those who had signed up but never actually completed a session it might encourage participants to attempt the first session. The second coaching session was scheduled for week four because we expected this would likely be a point where participants might be struggling with implementing eating and physical activity plans and feeling discouraged, but not yet have had several weeks without logging on (and therefore be more difficult to contact and engage).

Coaches made two initial attempts to telephone the participant to conduct the coaching. If they could not make contact in this way they sent an email, asking the participant to suggest a specific time for the call. If a response to this email was received they attempted a telephone call at the specified time.

If attempts to contact participants by telephone failed, the coach sent a supportive email with content largely corresponding to the verbal protocol and tailored to what was known about the participant (e.g. whether they had used the intervention so far, whether they had recorded a weight loss).

COACHING GUIDELINES

General Principles
The aim of these sessions are to encourage people to use the POWeR website, follow the advice and use the tools they find on it. Coaches can encourage POWeR users by being friendly and supportive and showing them that somebody cares about and notices their progress

Do's

1. **Do** be warm and friendly. The tone of the sessions is friendly, kind and supportive.

2. **Do** make the participant aware that you are keeping an eye on their progress/use of POWeR. We want them to think that somebody else cares about how they are getting on

3. **Do** praise any achievements and progress.

4. **Do** express confidence that POWeR works and that by following it people will lose weight gradually and safely and keep it off.

5. **Do** listen to the user and show that you understand any difficulties they talk about. Then gently encourage the user to continue to use the website.

6. **Do** accept that POWeR is not perfect/to everyone's taste but is worth continuing [if people discuss frustrating experiences with using POWeR]

7. **Do** keep language simple and positive. You can use similar language to the participant.

8. **Do** direct participants to their GP if they have serious health conditions or are very unsure about whether something is safe and appropriate and can't find out from POWeR.

Don'ts

1. **Don't** tell people what to do! Instead give them opportunities and prompts to come up with their own ideas of what they can do to solve any problems they've come across.

2. **Don't** offer advice! For example, **don't** give advice or tips on any specific diet, exercise, or other weight management method.

3. **Don't** worry about not being an expert on POWeR; it's a huge intervention with lots of different parts and you cannot possibly learn all the content.

4. **Don't** advise on any technical problems with the website (unless you are absolutely sure what's wrong). Make a brief note of the problem, apologise and say that the website is working well for most users but there are always a few bugs on websites. Say you will report the problem but that the technical team can't get back to POWeR users individually. Then email details to Leanne.

5. **Don't** spend too long on chat as time is limited, but some friendly conversation can help to build a good relationship

6. **Don't** allow the sessions to turn into a qualitative interview about their experiences of POWeR! If users try to give you feedback about it reassure them that we'll be really interested in getting in depth feedback later on, but that's not what coaching calls are for.

SESSION CHECKLIST

Session 1: What to cover	Tick
**Introduce self and role as coach**<br>Give your name. Explain you are trained to support people trying to manage weight using POWeR	
**Build a friendly relationship**<br>E.g. Thanks for taking time to talk, looking forward to supporting you with POWeR	
**Explain what you'll be doing**<br>A couple of brief phone calls to help you get the best from POWeR. Not specific advice. POWeR itself gives you skills/tools. I'll be checking in with you again in 4 weeks to see how things are going	
**Explain how you will keep an eye on their progress**<br>I'm able to log on and see your progress. We'll talk again and look at what you've achieved and talk through problems. OK?	
**Review POWeR use**<br>I've had a quick look at the website and I can see…….	
**Praise/encourage any POWeR use (e.g. logging on, setting goals).**<br>Well done. You've made an important step etc.	
**Ask about questions and concerns and point in direction of POWeR tools**<br>How's things been going with POWeR? Is there anything you've found hard, got stuck with? Can you think of anything to do about that? Have you had a look at what POWeR suggests?	
**Ask about goals and plans (if they've done a session) and be encouraging**<br>You decided to go for the goal about …….? You made a plan to ……….. How is that going?	
**Remind about ongoing support [Remind of anything they've said they'll do]-**<br>That's it for our first call. Look forward to speaking to you again in around 4 weeks.<br>[By that point you said you would……….. so we can talk through how that's gone].	
**Wrap up and end call**<br>Nice talking to you. Hope you achieve your goals.	
**Session 2: what to cover**	**Tick**
**Build a friendly relationship**<br>E.g. thanks for taking time to talk, nice to talk to you again	
**Reason for today's call**<br>You've been on the POWeR plan a little while now. I'm phoning to see how you are getting on.	
**Review POWeR use**<br>By logging onto the website and I can see…….	
**Praise/encourage any POWeR use (e.g. logging on, setting goals). If relevant, congratulate on weight loss**<br>You made a fantastic start with session 1 and 2. But you haven't done anything on POWeR since 3 weeks ago?	
**Ask about questions and concerns and point in direction of POWeR tools**<br>How's things been going with POWeR? Is there anything you've struggled with, got stuck with? Can you think of anything to do about that? Have you had a look at what POWeR suggests?	
**Ask about goals and plans (if they've done a session) and offer encouragement**<br>You decided to go for the goal about …….? You made a plan to ………..? How is that going?	
**Mention ongoing support [Remind them of anything they've said they will do]**<br>This is the last phone call you'll have from me. If you've found it helpful to have someone to talk to maybe think about friends/family…Over the next few weeks you said you would ….	
**Wrap up**<br>Well done for progress made so far. Good luck.	

DETAILED CONTENT/SCRIPTS FOR SESSIONS

Session 1: 10-15 minutes

**Details/script**	
Introduce self.<br><br>Build a friendly relationship	*Welcome to the POWeR program. Thank you for finding the time to talk to me.*<br><br>*I'm _____, I've been trained to help support people lose weight on the POWeR programme]. I will be working as your coach while you use POWeR. I look forward to supporting you with managing your weight.*
Explain what you'll be doing	*The phone calls with me will be brief- about 10 or 15 minutes*<br><br>*As your coach I will be supporting you in learning how to use the POWeR program well. But I won't be providing specific advice on what to eat or what exercise to do.*<br><br>*The POWeR program itself will give you the skills to manage your weight, not me. The sessions train you to be your own coach by giving you information and tools.*<br><br>*I'm here to support you in your use of POWeR.*<br><br>*The plan is that I will be checking in with you again in 4 weeks time to see how things are going with the program.*
Explain how you will keep an eye on their progress	*Over the next few weeks I'll be able to log on and see your progress online. So I'll be able to look and see whether you've logged in and done sessions and whether you've had any success with weight loss.*<br><br>*When we talk again, we'll look at any success you've had but also chat through any difficulties you've been having with the POWeR programme. Most people find POWeR fairly easy to use, but if you're having problems I'll support you with sorting them out.*<br><br>*Does that sound OK?*<br><br>**[If needed – reassure***:* *I won't be telling you off if you don't manage to do things, or making you to do the programme if you don't want to. I'll be here to encourage and support you with meeting your own goals and will help you to get the most out of the POWeR programme*.]
Review POWeR use<br><br><br><br>Praise and encourage any POWeR use	*I've already had a quick look on the website and it looks like…………………………*<br>**[Summarise what you can see about their use of POWeR and any success so far]**<br><br>**Congratulate the user on ANY way they have used the POWER programme so far. Find something positive to say.**<br>e.g. if they have completed a session, logged on, set goals, made plans, scheduled this call, made a commitment to do session 1 at a specific time, signed up to use POWeR, bought some scales for weight monitoring…..<br><br>"*I can see you've logged on and completed session 1- including choosing a plan to follow and making some goals and a reasons to lose weight card- that's great*"

	OR<br><br>*"You've made a positive step towards weight loss in signing up to do this and making the time to talk to me today. I can see that you haven't logged on and done session 1 yet though*? **[After they reply encourage them to log on. Link this into next section where appropriate]**
Ask about questions and concerns and point in direction of POWeR tools<br><br><br><br><br><br><br><br><br><br><br><br><br><br><br><br>Ask about goals and plans and offer encouragement	**Ask about /follow up on any problems or concerns mentioned.**<br>**e.g.**<br>• **Not had chance**<br>• **Not been motivated to log in**<br>• **Not sure about plans or goals they have set**<br>• **Didn't understand something**<br><br>**If there are concerns/questions ask participant what solutions they would like to try**<br><br>*I think it's great that you've been able to identify that as being a problem/barrier……..*<br>*Can you think of something you can do to get around that?*<br>*"Have you had another look at X?"*<br><br>**Get a commitment from them for a course of action but don't tell them what to do**<br><br><br>**If no concerns…. ask about goals and plans. Offer encouragement.**<br><br>*You decided to go for the goal about cutting out fizzy drinks? You made a plan to swap these for sugar free squash and to take a flask to work. How is that going?*<br><br>**[Well]** *It sounds like a good plan- I hope it goes well trying to put it into practice.*<br><br>**[Not so well]** *OK, when you next log back in you can review how you have done and think about if you want to change your goals or plans. The POWeR site gives you lots of opportunities to find what works best for you.*
Remind them of anything they've said they will do<br><br><br>Remind about ongoing support<br><br><br>Wrap up	*So, that's it for our first call. It was really nice talking to you.*<br><br>*Over the next few weeks you'll log in and do the online sessions* [if relevant, refer to any commitment they have made]. *Each of these teaches you new things and offers new tools to help you to lose weight.*<br><br>*I'll be keeping an eye on how things are going and we'll talk again in a few weeks when you've done some more of POWeR.*<br><br>*Hope it all goes well [participant's name] - I look forward to talking to you again*<br><br>**End call**

Session 2: 10 minutes

<table>
<tr><td colspan="2">Details/script</td></tr>
<tr><td>Greeting/building friendly relationship</td><td>Greet participant.</td></tr>
<tr><td>Reason for today's call</td><td>It's been 4 weeks since we last spoke, and you've been on the POWeR plan a little while now.

The reason I'm calling is to see how you are getting on and talk through any problems or questions you might have.

How does that sound?</td></tr>
<tr><td>Review POWeR use

Praise/encourage any POWeR use and/or weight loss</td><td>By logging on to POWeR as your coach I can see that {describe use/success on POWeR to date}

Congratulate the user on ANY aspect of using the POWeR programme so far.

Find something positive to say.

Congratulate on weight loss if there is any but pay attention mostly to their use of the web intervention.

E.g. if they have completed sessions, met goals, but not lost weight …..

"I can see you've logged on and done session 1, 2 and 3. You haven't lost any weight yet but you haven't gained any either. You should be proud of yourself – you have made a really positive step towards supporting long term, gradual weight loss"

If user is disappointed stress there it's normal for people to have good weeks and bad weeks. The most important thing is they are learning skills and strategies to lose weight and keep it off. [move onto next section about questions/concerns]

If they have not lost weight and have not completed sessions

"You made a fantastic start with session 1 and 2. But you haven't done anything on POWeR since 3 weeks ago?

[Give them chance to respond. Gently encourage them to keep going with POWeR. Link this into next section where appropriate]</td></tr>
<tr><td>Ask about questions and concerns and point in direction of POWeR tools</td><td>Ask about or follow up on any problems or concerns they have mentioned.
e.g.
• Not had chance, not been motivated to log in
• Not sure about plans or goals they have set
• Didn't understand something

If there are concerns/questions ask participant what solutions they would like to try

I think it's great that you've been able to realise that's a problem/barrier……..
Can you think of something you can do to get around that?
Have you had another look at session X?</td></tr>
</table>

Ask about goals and plans and offer encouragement	*Can you think of which POWeR tools might be able to help?*<br><br>**Try to encourage them to say what they want to/plan to do- but don't tell them what to do**<br><br>**If no concerns --- ask about recently-set goals and plans and strategies and tools being used. Offer encouragement.**<br><br>[Well] *It sounds like a good plan- I hope it goes well trying to put it into practice.*<br><br>[Not so well]- *When you next log back in you can review how you have done and think about if you want to change your goals or plans. The POWeR site gives you plenty of chances to find what works best for you.*
**[**Remind them of anything they've said they will do**]**<br><br>Mention ongoing support<br><br>Wrap up	*Over the next few weeks you'll keep logging in and doing the online sessions.* ***[refer to any commitment they have made]***<br><br>*This is the last phone call you'll have from me though. If you've found it helpful to have someone to talk to about being on the POWeR plan, maybe you can think of friends or family members who you can talk to about how things are going?*<br><br>*Good luck.*<br><br>**End call**
