# Supplementary material for: Does Brief Telephone Support Improve Engagement With a Web-Based Weight Management Intervention? Randomized Controlled Trial
Source: J Med Internet Res. 2014 Mar 28;16(3):e95. doi: 10.2196/jmir.3199 (PMC4004138; doi:10.2196/jmir.3199)
Supplement: Supplementary file 3 [file jmir_v16i3e95_app3.pdf]

CONSORT-EHEALTH Checklist V1.6.2 Report

(based on CONSORT-EHEALTH V1.6), available at [http://tinyurl.com/consort-ehealth-v1-6].

3199

Date completed
12/13/2013 10:56:02
by
Laura Dennison

Does brief telephone support improve engagement with a web-based weight management intervention? A randomised controlled trial

TITLE

1a-i) Identify the mode of delivery in the title
YES THIS ITEM IS ADDRESSED

"Does brief telephone support improve engagement with a web-based weight management intervention? A randomized controlled trial"

1a-ii) Non-web-based components or important co-interventions in title
YES THIS ITEM IS ADDRESSED

Does brief telephone support improve engagement with a web-based weight management intervention? A randomized controlled trial

1a-iii) Primary condition or target group in the title
YES THIS ITEM IS ADDRESSED

Does brief telephone support improve engagement with a web-based weight management intervention? A randomized controlled trial

ABSTRACT

1b-i) Key features/functionalities/components of the intervention and comparator in the METHODS section of the ABSTRACT
YES THIS ITEM IS ADDRESSED

"POWeR consists of weekly online sessions which emphasise self-monitoring, goal-setting, and cognitive/behavioral strategies".

"telephone coaching phone calls aimed at promoting continued usage of the website"

1b-ii) Level of human involvement in the METHODS section of the ABSTRACT
YES THIS ITEM IS ADDRESSED

"Our primary outcome was intervention usage and we investigated whether this was enhanced by the addition of brief telephone coaching."

"786 adults were randomized at an individual level through an online procedure to a) POWeR only (n=264) b) POWeR plus coaching (n=247)"

"Those in the POWeR plus coaching arm were contacted at approximately 7 and 28 days after randomisation for short telephone coaching phone calls aimed at promoting continued usage of the website. "

1b-iii) Open vs. closed, web-based (self-assessment) vs. face-to-face assessments in the METHODS section of the ABSTRACT
YES THIS ITEM IS ADDRESSED

"Participants were recruited using a range of methods including targeted mailouts, adverts in the local press, notices on organisational websites and social media. "

"Website usage was tracked automatically. Weight was assessed by online self-report. "

1b-iv) RESULTS section in abstract must contain use data
YES THIS ITEM IS ADDRESSED

"Of the 511 participants allocated to the two intervention groups the median number of POWeR sessions completed was just one (IQR for POWeR only= 0-2, IQR=0-3 for POWeR plus coach). Nonetheless, a substantial minority completed at least the core three sessions of POWeR; 47 participants (17.8%) in the POWeR only arm and 64 participants (25.9%) in the POWeR plus coach arm. In the primary outcome analysis, participants in the POWeR plus coaching group persisted with the intervention for longer. They were 1.61 times more likely to complete the three core sessions than the POWeR only group (2 (1, n=511)=4.93, OR =1.61, 95%CI=1.06-2.47)."

1b-v) CONCLUSIONS/DISCUSSION in abstract for negative trials
YES THIS ITEM IS ADDRESSED

"In common with most web-based interventions studies usage of POWeR was sub-optimal overall. Our findings suggest that supplementing web-based weight management with brief human support might improve usage and outcomes. However, uptake of telephone support may be low overall, with particular types of users more likely to engage with it."

INTRODUCTION

2a-i) Problem and the type of system/solution
YES THIS ITEM IS ADDRESSED

In the current study we disseminated ‘POWeR’, a completely automated web-based weight management intervention (described in detail below). Other RCTs (ISRCTN31685626 and ISRCTN21244703) are examining the efficacy of POWeR for weight loss in a primary care setting with nurse support. In contrast, the current study sought to investigate engagement with this intervention in a high reach, low cost public health context. Unlike previous web-based weight management trials our research procedures were handled automatically by our intervention software, which meant that the trial took place without participants having contact with the researchers at registration, baseline and follow-up. We examined engagement with the intervention and self-reported weight change in this more remote context and tested whether the provision of brief human support influenced this.

2a-ii) Scientific background, rationale: What is known about the (type of) system

YES THIS ITEM IS ADDRESSED

This subitem is addressed throughout the introduction section

"Internationally, obesity is one of the biggest public health concerns [1]. Interventions that promote changes in diet and physical activity and include behaviour modification techniques such as goal setting and self-monitoring are considered the gold standard of treatment [2]. However, high cost and low access limits the reach of such programmes when delivered face-to-face by health professionals [3,4]. The internet has emerged as a promising way to reach greater numbers of individuals at low cost and, in recent years, various web-based weight loss programmes have been developed and evaluated.
Despite holding promise as potentially cost-effective interventions, recent reviews of web-based weight loss interventions have found that effect sizes for weight loss tend to be fairly modest, with substantial heterogeneity in outcomes, and many online programmes suffer from suboptimal engagement [5,6]. Such findings are not limited to web-based weight loss interventions but are common across different types of e-health interventions.
One possible explanation for variations in the efficacy of and engagement with web- based interventions is the variations in the human contact participants have to support them as they participate in the web-based programme. Human support may be in various formats including face-to-face individual or group meetings, telephone calls, text messages, emails, or online chat. It may be from health professionals, researchers or technicians and may serve various purposes ranging from answering technical queries, to encouraging prolonged use, to providing substantial therapeutic input. Taken as a whole, the e-health literature suggests that engagement and behavioural or health outcomes for web-based interventions tend to be better when usage is accompanied by some form of human contact [7,8]. For example, within the web-based mental health literature, meta-analyses show larger effect sizes for interventions that also include some contact with a therapist than interventions that are wholly web-based [9,10] and qualitative studies suggest some participants perceive a need for human contact and support [11,12]. In the field of physical health and weight management, reviews have identified contact, counselling and support from a health professional as key elements responsible for high engagement and effectiveness of web-based interventions [13, 14]. Interestingly though, the few randomized controlled trials (RCTs) of weight management programmes that have directly compared different types or intensities of human support have not always found evidence that higher support versions have superior engagement or weight-related outcomes [4,15,16]. Yet, in these trials, even participants in the ostensible minimally-supported website arms actually received considerable human contact and support, including initial orientation to the website and contact with the research team throughout the trial [4,15], and counsellor-facilitated online chatrooms [16]. Therefore these trials cannot provide clear comparisons between web-based interventions provided with and without human support.
Overall, the question of how to use human contact to boost engagement with web interventions remains under elucidated. Still, it is an important question since the considerable reach and low marginal cost per additional user are amongst the key proposed benefits of web-based interventions. Yet many web-based weight management interventions evaluated to date have featured face-to-face orientation sessions plus various forms of telephone, email, web chat or face-to-face contact with a health care professional or researcher during the intervention period [4,15-18]. By adding human support, the cost and reach benefits may be undermined because costs increase when staff are required, particularly if support is provided by highly trained professionals. Gaining a better understanding of what types of human contact boost engagement with web interventions, and investigating brief and low-cost formats for such support, is therefore of great importance. It is equally important to understand how human contact/support might influence engagement with and effectiveness of web interventions. A clear framework for this has been absent until recent theoretical work on ‘Supportive Accountability’ [7] which proposes that human support can enhance adherence to e-Health interventions through accountability to another person. Accountability is the expectation that an individual may be called upon to explain his or her actions to another person. The model hypothesises that successfully fostering ‘Supportive Accountability’ involves some human presence, either face-to-face, or remotely. The model considers progress monitoring to be central to fostering accountability and proposes ways in which to conduct this in an effective and acceptable way. A recent trial using telephone coaching based upon this model showed that this form of human support increased adherence to a web-based depression intervention [19].
Current study context and aims
In the current study we disseminated ‘POWeR’, a completely automated web-based weight management intervention (described in detail below). Other RCTs (ISRCTN31685626 and ISRCTN21244703) are examining the efficacy of POWeR for weight loss in a primary care setting with nurse support. In contrast, the current study sought to investigate engagement with this intervention in a high reach, low cost public health context. Unlike previous web-based weight management trials our research procedures were handled automatically by our intervention software, which meant that the trial took place without participants having contact with the researchers at registration, baseline and follow-up. We examined engagement with the intervention and self-reported weight change in this more remote context and tested whether the provision of brief human support influenced this.
Our primary aim was to assess whether human support in the form of brief telephone coaching, based around the Supportive Accountability framework [7], improved engagement with our web-based weight intervention, as measured by session usage. Secondary analyses examined the self-reported weight loss that participants experienced when following the coached and uncoached version of the intervention. We also explored the uptake of telephone coaching, whether this was associated with user characteristics and outcomes, and whether accountability to a coach might be a mechanism through which coaching boosts engagement with the website and weight loss."

METHODS

3a) CONSORT: Description of trial design (such as parallel, factorial) including allocation ratio
YES THIS ITEM IS ADDRESSED

"We examined engagement with the intervention and self-reported weight change in this more remote context and tested whether the provision of brief human support influenced this.
Our primary aim was to assess whether human support in the form of brief telephone coaching, based around the Supportive Accountability framework [7], improved engagement with our web-based weight intervention, as measured by session usage. Secondary analyses examined the self-reported weight loss that participants experienced when following the coached and uncoached version of the intervention. We also explored the uptake of telephone coaching, whether this was associated with user characteristics and outcomes, and whether accountability to a coach might be a mechanism through which coaching boosts engagement with the website and weight loss."

3b) CONSORT: Important changes to methods after trial commencement (such as eligibility criteria), with reasons
NOT IN MANUSCRIPT

There were no such changes to report or this study..

3b-i) Bug fixes, Downtimes, Content Changes
YES THIS ITEM IS ADDRESSED

During the trial the intervention content was ‘frozen’ and no changes or bug fixes were made to the POWeR website

4a) CONSORT: Eligibility criteria for participants

YES THIS ITEM IS ADDRESSED

"To proceed through the registration process participants had to report being UK resident adults with a body mass index (BMI) of ≥23 and having regular Internet access. Users were cautioned to consult a health professional prior to using POWeR if they reported having a condition that might make changing diet and exercise inappropriate."

4a-i) Computer / Internet literacy

YES THIS ITEM IS ADDRESSED

we state in eligibility criteria that participants had to report "having regular Internet access"

4a-ii) Open vs. closed, web-based vs. face-to-face assessments:

YES THIS ITEM IS ADDRESSED

"A variety of methods were used to recruit participants from community settings in the North East of the UK. This included mailed invitations to 15,000 homes, local press releases, posters in community settings, and information on local government and NHS public health websites and intranet, as well as paid advertising on Facebook and post/tweets on organisational social media. "

"All recruitment procedures including study information, eligibility screening, obtaining informed consent, baseline data collection and randomisation were conducted online using automated procedures."

"The first author's email address was provided for asking questions prior to signing up (no questions were received). A "POWeR" email address was provided once participants were in the trial. Brief email contact between participants and the first author took place if participants needed to report technical problems or request withdrawal or cancelling of automatic email prompts or reminders. With the exception of coach phone calls and emails received by the POWeR plus coaching participants, there was no other human contact with participants whilst they were in the trial."

"The primary outcome variable (engagement with the web-based intervention) was automatically logged by the intervention software. The LifeGuide software logs all usage data including which pages were viewed, in which order, when, and for how long"

"All self-report data were collected using web-based questionnaires."

4a-iii) Information giving during recruitment

YES THIS ITEM IS ADDRESSED

"Recruitment materials invited members of the public to try a new online weight management programme as part of a research trial. Recruitment materials and participant information sheets carried the organisation name and logo of the local NHS public health organisation and also emphasised the involvement of academics and clinicians from the University of Southampton in the development of the intervention."

4b) CONSORT: Settings and locations where the data were collected

YES THIS ITEM IS ADDRESSED

"A variety of methods were used to recruit participants from community settings in the North East of the UK."

4b-i) Report if outcomes were (self-)assessed through online questionnaires

YES THIS ITEM IS ADDRESSED

"The primary outcome variable (engagement with the web-based intervention) was automatically logged by the intervention software. The LifeGuide software logs all usage data including which pages were viewed, in which order, when, and for how long. For the current analyses we analysed the number of POWeR sessions each participant had completed by 8 week follow-up.
All self-report data were collected using web-based questionnaires."

4b-ii) Report how institutional affiliations are displayed

YES THIS ITEM IS ADDRESSED

"Recruitment materials invited members of the public to try a new online weight management programme as part of a research trial. Recruitment materials and participant information sheets carried the organisation name and logo of the local NHS public health organisation and also emphasised the involvement of academics and clinicians from the University of Southampton in the development of the intervention."

5) CONSORT: Describe the interventions for each group with sufficient details to allow replication, including how and when they were actually administered

5-i) Mention names, credential, affiliations of the developers, sponsors, and owners

YES THIS ITEM IS ADDRESSED

"....constructed using the LifeGuide open access intervention authoring software (www.lifeguideonline.org). "

"Conflict of interest
LY, PL,SW and KB were involved in the original development of the POWeR intervention. However this is not a commercial intervention. No other conflicts of interest are declared."

5-ii) Describe the history/development process

YES THIS ITEM IS ADDRESSED

"POWeR is a fully automated, tailored, web-based weight management intervention constructed using the LifeGuide open access intervention authoring software (www.lifeguideonline.org). The intervention aims to empower users to become their own personal health trainer through the development of new self-regulation skills. POWeR draws on various theoretical models and incorporate multiple behaviour change techniques. The intervention planning and considerable iterative qualitative work undertaken during development stages are described in detail elsewhere [21]. "

5-iii) Revisions and updating

YES THIS ITEM IS ADDRESSED

During the trial the intervention content was 'frozen' and no changes or bug fixes were made to the POWeR website

5-iv) Quality assurance methods

NOT ADDRESSED IN MANUSCRIPT

It is unclear what sort of quality assurance methods would be relevant for the current study. This e-CONSORT item would benefit from further elaboration/guidelines for authors.

5-v) Ensure replicability by publishing the source code, and/or providing screenshots/screen-capture video, and/or providing flowcharts of the algorithms used

YES THIS ITEM IS ADDRESSED

"A demonstration version of POWeR can be accessed at https://www.lifeguideonline.org/player/play/powernedemo"

A screenshot is also included as an appendix.

5-vi) Digital preservation

YES THIS ITEM IS ADDRESSED

NB- The URL used in the study will not continue to work. See item 5-v for how to see more about the POWeR content.

5-vii) Access

YES THIS ITEM IS ADDRESSED

"A variety of methods were used to recruit participants from community settings in the North East of the UK. This included mailed invitations to 15,000 homes, local press releases, posters in community settings, and information on local government and NHS public health websites and intranet, as well as paid advertising on Facebook and post/tweets on organisational social media. Participation was free and no financial incentives were provided."

"All recruitment procedures including study information, eligibility screening, obtaining informed consent, baseline data collection and randomisation were conducted online using automated procedures."

5-viii) Mode of delivery, features/functionalities/components of the intervention and comparator, and the theoretical framework

YES THIS ITEM IS ADDRESSED

"POWeR is a fully automated, tailored, web-based weight management intervention constructed using the LifeGuide open access intervention authoring software (www.lifeguideonline.org). The intervention aims to empower users to become their own personal health trainer through the development of new self-regulation skills. POWeR draws on various theoretical models and incorporate multiple behaviour change techniques. The intervention planning and considerable iterative qualitative work undertaken during development stages are described in detail elsewhere [21]. POWeR is structured as a series of online sessions. In session one, users choose an eating plan, explore their personal motivations for weight loss, and set personalised eating goals to follow in the subsequent week. Further sessions all begin with a weight and goal review (i.e. self-monitoring and goal setting with tailored feedback) and then progress to new content which includes information and tools to help develop cognitive and behavioural self-regulatory weight management skills, each with an explicit scientific rationale. There are interactive activities for participants to complete, user stories/testimonials and optional links to more detailed information, including to reputable external websites. The second session theme is social support and the third focuses on physical activity. The first three sessions are core sessions that each user is tunnelled through; then from the fourth session onwards, after initially working through the weight and goal review, users have a choice of whether to also access web pages about specific topics that interest them (e.g emotional eating, fitting healthy behaviour into busy lives) or whether to end the session at that point. "

5-ix) Describe use parameters

YES THIS ITEM IS ADDRESSED

"I"Intended use of POWeR is the completion of one session per week. Each time a session is completed the subsequent session becomes available 7 days later and remains available until the user next logs in. Participants receive automatic email reminders to advise them that their new session is ready, provide a description of what will be covered, and invite them to log in to use it. They also received one automatic email reminder one week later if they did not log on. A total of 12 different sessions are available and users can continue to complete sessions for as long as they are finding it useful and can log in to complete weekly weight and goal reviews even after all sessions have been completed. In the current trial we followed up participants and examined their engagement with the intervention and weight loss eight weeks after randomisation"

5-x) Clarify the level of human involvement

YES THIS ITEM IS ADDRESSED

"All recruitment procedures including study information, eligibility screening, obtaining informed consent, baseline data collection and randomisation were conducted online using automated procedures".

.

"The first author's email address was provided for asking questions prior to signing up (no questions were received). A "POWeR" email address was provided once participants were in the trial. Brief email contact between participants and the first author took place if participants needed to report technical problems or request withdrawal or cancelling of automatic email prompts or reminders. With the exception of coach phone calls and emails received by the POWeR plus coaching participants, there was no other human contact with participants whilst they were in the trial."

"The coaching calls aimed to promote continued usage of the POWeR website and adherence to the recommendations within the website. Coaching procedures were developed based on the Supportive Accountability model [7]. Coaches could access a coaching portal of POWeR where they were able to review the usage patterns of participants, a graph showing weight change, and the participant's current eating and physical activity goals and plans. Coaching sessions were focussed on promoting ongoing use of the web intervention by monitoring usage and giving feedback on progress, offering support and encouragement for use of the website. Each coaching call was intended to last for approximately 10 minutes. Coaches followed detailed protocols (see Electronic Supplementary Materials) which set out ways to proceed with the two phone calls depending on whether the user was engaging with POWeR as intended. Table 1 summarises key features of the calls."

5-xi) Report any prompts/reminders used

YES THIS ITEM IS ADDRESSED

"Participants receive automatic email reminders to advise them that their new session is ready, provide a description of what will be covered, and invite them to log in to use it. They also received one automatic email reminder one week later if they did not log on."

5-xii) Describe any co-interventions (incl. training/support)
YES THIS ITEM IS ADDRESSED

"The coaching calls aimed to promote continued usage of the POWeR website and adherence to the recommendations within the website. Coaching procedures were developed based on the Supportive Accountability model [7]. Coaches could access a coaching portal of POWeR where they were able to review the usage patterns of participants, a graph showing weight change, and the participant's current eating and physical activity goals and plans. Coaching sessions were focussed on promoting ongoing use of the web intervention by monitoring usage and giving feedback on progress, offering support and encouragement for use of the website. Each coaching call was intended to last for approximately 10 minutes. Coaches followed detailed protocols (see Electronic Supplementary Materials) which set out ways to proceed with the two phone calls depending on whether the user was engaging with POWeR as intended. Table 1 summarises key features of the calls. "

"The coaches were eight postgraduate students and research assistants affiliated with the health psychology research centre at the University of Southampton. Assignment to a particular coach was on the basis of coach workload/availability. With one exception (where the coach left the institution), all participants had the same coach for both calls. Coaches completed approximately 3.5 hours of training each. This included working through the core sessions of POWeR themselves and attending a training session which covered trial procedures, the POWeR intervention philosophy and the coaching protocol. Coaches were closely supervised throughout the trial period by the first author who listened to audio recordings of the coaching sessions and used a brief checklist to help monitor and ensure fidelity to protocol.
Following randomisation, participants in the coach arm were shown a web page advising them that they were going to be contacted by a POWeR coach who would provide them with support in following the programme. The web page allowed participants to suggest optimal times/days for being contacted (limited to weekdays 8am to 8pm) and provide details of the best phone numbers for coaches to contact them on. Attempts to contact participants for their coaching calls were made at the end of week 1 and the end of week 4. Coaches made two initial attempts to telephone the participant to conduct the coaching. If they could not make contact in this way they sent an email, asking the participant to suggest a specific time for the call. If a response to this email was received they attempted a telephone call at the specified time. If attempts to contact participants by telephone failed, the coach sent a supportive email with content largely corresponding to the verbal protocol and tailored to what was known about the participant (e.g. whether they had used the intervention so far, whether they had recorded a weight loss)."

6a) CONSORT: Completely defined pre-specified primary and secondary outcome measures, including how and when they were assessed
YES THIS ITEM IS ADDRESSED

The primary outcome variable (engagement with the web-based intervention) was automatically logged by the intervention software. The LifeGuide software logs all usage data including which pages were viewed, in which order, when, and for how long. For the current analyses we analysed the number of POWeR sessions each participant had completed by 8 week follow-up.

"At baseline[.....]Participants self-reported height (in cm or feet and inches) and weight (as measured on home scales)."
"At follow-up participants in all treatment arms were asked to enter their current weight "
6a-i) Online questionnaires: describe if they were validated for online use and apply CHERRIES items to describe how the questionnaires were designed/deployed
YES THIS ITEM IS PARTIALLY ADDRESSED

"All self-report data were collected using web-based questionnaires. To ensure we had complete data on our participants at baseline, all baseline questionnaires were mandatory (i.e. the participant could not progress without submitting a response). The follow-up point was 8 weeks post-randomisation. Automatically-generated emails requested participants to complete a brief follow-up questionnaire and included a hyperlink to web-based questionnaires. Up to three reminder emails were automatically issued after 5, 10 and 15 days of non-response."
6a-ii) Describe whether and how "use" (including intensity of use/dosage) was defined/measured/monitored
YES THIS ITEM IS ADDRESSED

"The primary outcome variable (engagement with the web-based intervention) was automatically logged by the intervention software. The LifeGuide software logs all usage data including which pages were viewed, in which order, when, and for how long. For the current analyses we analysed the number of POWeR sessions each participant had completed by 8 week follow-up."
6a-iii) Describe whether, how, and when qualitative feedback from participants was obtained
NOT ADDRESSED

Our qualitative sub-study will be a secondary publication. It is not mentioned here as the qualitative data collection and analysis does not correspond to the aims/research questions being addressed here.
6b) CONSORT: Any changes to trial outcomes after the trial commenced, with reasons
YES THIS ITEM IS ADDRESSED

"For the primary analysis we planned to conduct independent t tests to examine between-arm differences in mean number of sessions completed. However, due to highly skewed data, such analysis was inappropriate. Instead, and as recommended by Glasgow et al. [15], we computed a meaningful 'threshold' usage dichotomous variable which indicated whether or not the participant had completed the core three POWeR sessions. Between arm differences were then analysed using a Chi square test"
7a) CONSORT: How sample size was determined
7a-i) Describe whether and how expected attrition was taken into account when calculating the sample size
YES THIS ITEM IS ADDRESSED

"The sample size was calculated a priori using GPOWER v3.1 [20]. To compare the primary outcome (intervention usage) in the POWeR only and POWeR plus coaching arm we calculated that we would need 253 participants in each arm to detect a small effect size (d=.25) with .80 power (alpha . 05, 2 tailed test). Therefore we aimed to randomize a total of 759 participants. It was not necessary to recruit additional participants to allow for attrition because our primary outcome was automatically logged by the website for all participants."
7b) CONSORT: When applicable, explanation of any interim analyses and stopping guidelines

ITEM NOT ADDRESSED

Not applicable- there were no interim analyses. The trial stopped when target sample size had been recruited.

8a) CONSORT: Method used to generate the random allocation sequence

YES THIS ITEM IS ADDRESSED

"All recruitment procedures including study information, eligibility screening, obtaining informed consent, baseline data collection and randomisation were conducted online using automated procedures"

"Randomisation was at the individual level and stratified by BMI (lower BMI <27.5 vs. higher BMI ≥27.5). Participants were allocated with a balanced ratio to one of three arms. "

8b) CONSORT: Type of randomisation; details of any restriction (such as blocking and block size)

YES THIS ITEM IS ADDRESSED

"Randomisation was at the individual level and stratified by BMI (lower BMI <27.5 vs. higher BMI ≥27.5). Participants were allocated with a balanced ratio to one of three arms. "

9) CONSORT: Mechanism used to implement the random allocation sequence (such as sequentially numbered containers), describing any steps taken to conceal the sequence until interventions were assigned

YES THIS ITEM IS ADDRESSED

"conducted online using automated procedures"

10) CONSORT: Who generated the random allocation sequence, who enrolled participants, and who assigned participants to interventions

YES THIS ITEM IS ADDRESSED

"All recruitment procedures including study information, eligibility screening, obtaining informed consent, baseline data collection and randomisation were conducted online using automated procedures."

"Randomisation was at the individual level and stratified by BMI (lower BMI <27.5 vs. higher BMI ≥27.5). Participants were allocated with a balanced ratio to one of three arms"

11a) CONSORT: Blinding - If done, who was blinded after assignment to interventions (for example, participants, care providers, those assessing outcomes) and how

11a-i) Specify who was blinded, and who wasn't

YES THIS ITEM IS ADDRESSED

"It was impossible to blind participants or coaches to trial arm assignment. Researchers were not blinded but did not interact with or collect data from participants directly as usage was tracked automatically and self-report data was collected via online questionnaires."

11a-ii) Discuss e.g., whether participants knew which intervention was the "intervention of interest" and which one was the "comparator"

YES THIS ITEM IS ADDRESSED

It was impossible to blind participants or coaches to trial arm assignment. Researchers were not blinded but did not interact with or collect data from participants directly as usage was tracked automatically and self-report data was collected via online questionnaires.

"Following randomisation, participants in the coach arm were shown a web page advising them that they were going to be contacted by a POWeR coach who would provide them with support in following the programme. "

11b) CONSORT: If relevant, description of the similarity of interventions

YES THIS ITEM IS ADDRESSED

Methods section describes differences between participants randomised to POWeR only and those randomised to POWeR plus coaching.

12a) CONSORT: Statistical methods used to compare groups for primary and secondary outcomes

YES THIS ITEM IS ADDRESSED

"All analyses were conducted in SPSS version 20. Means and standard deviations were computed for continuous variables, n and % were computed for categorical variables. We used an alpha level of .05 for all statistical tests. For the primary analysis we planned to conduct independent t tests to examine between-arm differences in mean number of sessions completed. However, due to highly skewed data, such analysis was inappropriate. Instead, and as recommended by Glasgow et al. [15], we computed a meaningful 'threshold' usage dichotomous variable which indicated whether or not the participant had completed the core three POWeR sessions. Between arm differences were then analysed using a Chi square test.
To examine between arm differences in self-reported weight loss we used ANCOVA, with follow-up weight as a DV, baseline weight as a covariate and trial arm as the IV. We performed an intention to treat (ITT) analysis using the last weight entry carried forward (from either baseline or as entered during intervention usage if follow-up was not completed). We also conducted a completers analysis by repeating another ANCOVA on the sample of participants who had completed follow-up measures. We also categorised participants according to whether or not they had lost at least 3 kilos at 8 week follow-up. Such weight loss would correspond to approximately 0.4 kilo (just under 1 pound) weight loss per week and would indicate a rate of weight loss in line with the POWeR programme recommendations which emphasizes building healthy habits rather than rapid weight loss. We reported the percentage of participants in each arm meeting this criterion".

12a-i) Imputation techniques to deal with attrition / missing values

YES THIS ITEM IS ADDRESSED

"We performed an intention to treat (ITT) analysis using the last weight entry carried forward (from either baseline or as entered during intervention usage if follow-up was not completed). We also conducted a completers analysis by repeating another ANCOVA on the sample of participants who had completed follow-up measures."

12b) CONSORT: Methods for additional analyses, such as subgroup analyses and adjusted analyses

YES THIS ITEM IS ADDRESSED

"To examine between arm differences in self-reported weight loss we used ANCOVA, with follow-up weight as a DV, baseline weight as a covariate and trial arm as the IV. We performed an intention to treat (ITT) analysis using the last weight entry carried forward (from either baseline or as entered during intervention usage if follow-up was not completed). We also conducted a completers analysis by repeating another ANCOVA on the sample of participants who had completed follow-up measures. We also categorised participants according to whether or not they had lost at least 3 kilos at 8 week follow-up. Such weight loss would correspond to approximately 0.4 kilo (just under 1 pound) weight loss per week and would indicate a rate of weight loss in line with the POWeR programme recommendations which emphasizes building healthy habits rather than rapid weight loss. We reported the percentage of participants in each arm meeting this criterion."

RESULTS

13a) CONSORT: For each group, the numbers of participants who were randomly assigned, received intended treatment, and were analysed for the primary outcome
YES THIS ITEM IS ADDRESSED

"Website usage patterns were analysed in the 511 participants allocated to the two active intervention arms"

13b) CONSORT: For each group, losses and exclusions after randomisation, together with reasons
YES THIS ITEM IS ADDRESSED

See CONSORT flow diagram

13b-i) Attrition diagram
YES THIS ITEM IS ADDRESSED

No diagram. Usage of the intervention is our primary outcome and is reported in results section.

14a) CONSORT: Dates defining the periods of recruitment and follow-up
YES THIS ITEM IS ADDRESSED

"A variety of methods were used to recruit participants from community settings in the North East of the UK between June 2012 and January 2013"

We state that follow-up was at 8 weeks after randomization.

14a-i) Indicate if critical “secular events” fell into the study period
NOT MENTIONED IN MANUSCRIPT
There were no such events to report.

14b) CONSORT: Why the trial ended or was stopped (early)
NOT MENTIONED IN MANUSCRIPT

There was no early ending to the trial. It stopped when sample size was met.

15) CONSORT: A table showing baseline demographic and clinical characteristics for each group
YES THIS ITEM IS ADDRESSED

See Table 2.

15-i) Report demographics associated with digital divide issues
YES THIS ITEM IS ADDRESSED

age, gender, education, deprivation index health literacy, hours of internet usage are reported in Table 2.

16a) CONSORT: For each group, number of participants (denominator) included in each analysis and whether the analysis was by original assigned groups

16-i) Report multiple “denominators” and provide definitions
YES THIS ITEM IS ADDRESSED

Addressed throughout results section

N is reported for each analysis and in each table. Analyses are clearly labelled as being either intention to treat or involving specific subgroups.

16-ii) Primary analysis should be intent-to-treat
YES THIS ITEM IS ADDRESSED

"our primary outcome was automatically logged by the website for all participants"

"Website usage patterns were analysed in the 511 participants allocated to the two active intervention arms. "

17a) CONSORT: For each primary and secondary outcome, results for each group, and the estimated effect size and its precision (such as 95% confidence interval)
YES THIS ITEM IS ADDRESSED

"Those in the POWeR plus coach arm were 1.61 times (95%CI=1.06-2.47) more likely to have continued to use POWeR until at least the end of the core 3 sessions (2 (1, n=511) =4.93, p=.026)."

"In the ITT analysis between arm differences were significant (F (2) =12.42, p<.001). Post-hoc pairwise comparisons indicated that those in the coach arm lost more weight than those in the control arm (mean difference = .915 kilos, p<.001, d=-.43, 95%CI=-.61 to-.26). Those in the POWeR only arm also lost more weight than those in the control arm (mean difference =.564 kilos, p<.001, d=-.27 95%CI=-.44 to-.10). The difference between the POWeR plus coaching and the POWeR only arms did not quite reach significance (mean difference .350 kilos, p=.063, d=-.17, 95%CI=-.34 to .01). "

17a-i) Presentation of process outcomes such as metrics of use and intensity of use
YES THIS ITEM IS ADDRESSED

Usage is our primary outcome and is reported in the results section.

17b) CONSORT: For binary outcomes, presentation of both absolute and relative effect sizes is recommended

YES THIS ITEM IS ADDRESSED

"a substantial minority completed at least the core three sessions of POWeR; 47 participants (17.8%) in the POWeR only arm and 64 participants (25.9%) in the POWeR plus coach arm. Those in the POWeR plus coach arm were 1.61 times (95%CI=1.06-2.47) more likely to have continued to use POWeR until at least the end of the core 3 sessions (2 (1, n=511) =4.93, p=.026)."

18) CONSORT: Results of any other analyses performed, including subgroup analyses and adjusted analyses, distinguishing pre-specified from exploratory

YES THIS ITEM IS ADDRESSED

Exploratory and subgroup analyses are labelled as such.

See section entitled "exploring coaching uptake"

18-i) Subgroup analysis of comparing only users

NOT APPLICABLE

Analyses of only website users are not presented. We present ITT analyses plus additional analyses based on participants who provided follow-up data.

19) CONSORT: All important harms or unintended effects in each group

NOT APPLICABLE

There were no harms or unintended effects to report.

19-i) Include privacy breaches, technical problems

NOT APPLICABLE

We had no privacy breaches and no technical problems of relevance to the analyses we are reporting.

19-ii) Include qualitative feedback from participants or observations from staff/researchers

NOT APPLICABLE

A qualitative study will be published separately.

DISCUSSION

20) CONSORT: Trial limitations, addressing sources of potential bias, imprecision, multiplicity of analyses

20-i) Typical limitations in ehealth trials

YES THIS ITEM IS ADDRESSED

"Strengths and limitations
A strength of this research is that our coaching protocols were well documented, specific in their aims and based on a theoretical model of engagement with digital interventions. Such explicit explanation of the aims and nature of human contact is rare in the reporting of web-based interventions. Furthermore the coach contact was brief (around 15 minutes for participants receiving the full dose) and delivered by providers with minimal training. This type of additional human support should be replicable in future studies and might prove feasible to implement and cost-effective for improving engagement and boosting intervention effectiveness even if effect sizes are modest.
The current study benefited from having primary outcome data available from all participants (by automatically tracking website usage). However, there was very high loss to follow-up for the self-reported questionnaires which means that the secondary analyses based on these follow-up measures need to be interpreted with caution. Nevertheless, our ITT analysis strategy bringing forward last recorded weight included all randomized participants. Due to the large number and wide geographical dispersion of participants, only self-reported weight data could be obtained. Most web-based weight loss trials have obtained objective weight data at face-to-face baseline and follow-up assessments [16-18,26,27]. However, a recent study suggests that online self-reported weight tends to be reasonably accurate [28] and given that testing the efficacy of POWeR for weight loss was not our main research question, we believe the self-report data is a useful initial indicator of likely weight loss from using POWeR in the short term. Furthermore, the absence of face-to-face assessment in this study allowed us to obtain website usage data in a context where there is no contact with a researcher. We believe that weight loss, website usage and retention for follow-up in many existing web weight loss trials may be influenced to some degree by the contact with researchers, expectation of being weighed by the research team at a later date, and perceptions of accountability and pressure this creates. In the current study, we may have obtained a more representative view of users, usage patterns and weight loss in contexts similar to what could be practical and affordable for a public health intervention."

21) CONSORT: Generalisability (external validity, applicability) of the trial findings

21-i) Generalizability to other populations

YES THIS ITEM IS ADDRESSED

"This type of additional human support should be replicable in future studies and might prove feasible to implement and cost-effective..."

"the absence of face-to-face assessment in this study allowed us to obtain website usage data in a context where there is no contact with a researcher. We believe that weight loss, website usage and retention for follow-up in many existing web weight loss trials may be influenced to some degree by the contact with researchers, expectation of being weighed by the research team at a later date, and perceptions of accountability and pressure this creates. In the current study, we may have obtained a more representative view of users, usage patterns and weight loss in contexts similar to what could be practical and affordable for a public health intervention."

21-ii) Discuss if there were elements in the RCT that would be different in a routine application setting

YES THIS ITEM IS ADDRESSED

"In the current study, we may have obtained a more representative view of users, usage patterns and weight loss in contexts similar to what could be practical and affordable for a public health intervention."

22) CONSORT: Interpretation consistent with results, balancing benefits and harms, and considering other relevant evidence

22-i) Restate study questions and summarize the answers suggested by the data, starting with primary outcomes and process outcomes (use)

YES THIS ITEM IS ADDRESSED

See discussion sections: "Engagement with the POWeR intervention and self-reported weight loss " Uptake of telephone coaching" and "Supportive Accountability as a mechanism for boosting engagement"

22-ii) Highlight unanswered new questions, suggest future research

YES THIS ITEM IS ADDRESSED

See discussion sections: "Engagement with the POWeR intervention and self-reported weight loss " Uptake of telephone coaching" and "Supportive Accountability as a mechanism for boosting engagement" Strengths and limitations"

Other information

23) CONSORT: Registration number and name of trial registry

YES THIS ITEM IS ADDRESSED

Abstract; "Trial registration: ISRCTN98176068 "

Method "the trial was registered with www.controlledtrials.com (ISRCTN98176068)."

24) CONSORT: Where the full trial protocol can be accessed, if available

YES THIS ITEM IS ADDRESSED

"the trial was registered with www.controlledtrials.com (ISRCTN98176068)."

The controlledtrials website provides an outline of the study. We have not published a full trial protocol.

25) CONSORT: Sources of funding and other support (such as supply of drugs), role of funders

YES THIS ITEM IS ADDRESSED

"This study was conducted with the support of a grant from the Engineering and Physical Sciences Research Council (EP/I032673/1: UBhave: ubiquitous and social computing for positive behaviour change). "

X26-i) Comment on ethics committee approval

YES THIS ITEM IS ADDRESSED

"Ethics and research governance approvals were granted by the University of Southampton "

x26-ii) Outline informed consent procedures

YES THIS ITEM IS ADDRESSED

"All recruitment procedures including study information, eligibility screening, obtaining informed consent, baseline data collection and randomisation were conducted online using automated procedures. "

X26-iii) Safety and security procedures

YES THIS ITEM IS ADDRESSED

"Users were cautioned to consult a health professional prior to using POWeR if they reported having a condition that might make changing diet and exercise inappropriate."

T"he first author's email address was provided for asking questions prior to signing up (no questions were received). A "POWeR" email address was provided once participants were in the trial. "

X27-i) State the relation of the study team towards the system being evaluated

YES THIS ITEM IS ADDRESSED

"Conflict of interest:
LY, PL, SW and KB were involved in the original development of the POWeR intervention. However this is not a commercial intervention. No other conflicts of interest are declared."
